# Biomedical Composites of Polycaprolactone/Hydroxyapatite for Bioplotting: Comprehensive Interpretation of the Reinforcement Course

**DOI:** 10.3390/polym16172400

**Published:** 2024-08-24

**Authors:** Markos Petousis, Nikolaos Michailidis, Apostolos Korlos, Vassilis Papadakis, Constantine David, Dimitrios Sagris, Nikolaos Mountakis, Apostolos Argyros, John Valsamos, Nectarios Vidakis

**Affiliations:** 1 Department of Mechanical Engineering, Hellenic Mediterranean University, 71410 Heraklion, Greece; markospetousis@hmu.gr (M.P.); mountakis@hmu.gr (N.M.); valsamos@hmu.gr (J.V.); 2 Physical Metallurgy Laboratory, Mechanical Engineering Department, School of Engineering, Aristotle University of Thessaloniki, 54124 Thessaloniki, Greece; nmichail@auth.gr (N.M.); aargyros@auth.gr (A.A.); 3 Centre for Research & Development of Advanced Materials (CERDAM), Center for Interdisciplinary Research and Innovation, Balkan Centre, Building B’, 10th km Thessaloniki-Thermi Road, 57001 Thessaloniki, Greece; 4 Department of Industrial Engineering and Management, International Hellenic University, 14th km Thessaloniki—N. Moudania, Thermi, 57001 Thessaloniki, Greece; apkorlos@ihu.gr; 5 Department of Industrial Design and Production Engineering, University of West Attica, 12243 Athens, Greece; v.papadakis@uniwa.gr; 6 Foundation for Research and Technology Hellas (FORTH), Institute of Electronic Structure and Laser (IESL), 70013 Heraklion, Greece; 7 Department of Mechanical Engineering, International Hellenic University, Serres Campus, 62124 Serres, Greece; david@ihu.gr (C.D.); dsagris@ihu.gr (D.S.)

**Keywords:** polycaprolactone (PCL), hydroxyapatite (HAp), fused filament fabrication (FFF), three-dimensional bio-plotting, scanning electron microscopy (SEM), micro-computed tomography (μCT)

## Abstract

Robust materials in medical applications are sought after and researched, especially for 3D printing in bone tissue engineering. Poly[ε-caprolactone] (PCL) is a commonly used polymer for scaffolding and other medical uses. Its strength is a drawback compared to other polymers. Herein, PCL was mixed with hydroxyapatite (HAp). Composites were developed at various concentrations (0.0–8.0 wt. %, 2.0 step), aiming to enhance the strength of PCL with a biocompatible additive in bioplotting. Initially, pellets were derived from the shredding of filaments extruded after mixing PCL and HAp at predetermined quantities for each composite. Specimens were then manufactured by bioplotting 3D printing. The samples were tested for their thermal and rheological properties and were also mechanically, morphologically, and chemically examined. The mechanical properties included tensile and flexural investigations, while morphological and chemical examinations were carried out employing scanning electron microscopy and energy dispersive spectroscopy, respectively. The structure of the manufactured specimens was analyzed using micro-computed tomography with regard to both their dimensional deviations and voids. PCL/HAp 6.0 wt. % was the composite that showed the most enhanced mechanical (14.6% strength improvement) and structural properties, proving the efficiency of HAp as a reinforcement filler in medical applications.

## 1. Introduction

The method of additive manufacturing (AM) is capable of fabricating objects by utilizing several materials. The material can be in powder, liquid, or solid form. The production of the final object is based on the previously designed 3D model, as well as the chosen process parameters [1]. AM methods possess some advantages of great importance, which are also the reason for their utilization in the first place. These include the ability to create parts of high complexity, cost-effectiveness, ease of controlling the morphology and size of pores, and simplicity [2,3]. In the medical field, using specially developed medical-grade polymers such as polypropylene (PP) [4], polyamide 12 (PA12) [5,6,7], and polylactic acid (PLA) [8,9] in material extrusion (MEX) 3D printing, and biomedical resins in vat photopolymerization is widely applied and researched [10,11]. Additives are used to improve the performance and induce antibacterial or other properties, such as silver [6], copper [7], ceramics [4], or biocompatible nature-sourced additives, such as cellulose [12,13].

Polycaprolactone (PCL) is a synthetic polymer [14]. Its degradation can occur either through bulk erosion or hydrolysis when its melting temperature is low [15]. It is usually employed in applications related to tissue engineering and regenerative medicine [16,17,18], because of the suitability of its properties. It is biocompatible, biodegradable, and processable because of its low melting temperature, rheological, viscoelastic properties, and thermal stability [19,20,21]. Additional properties include stiffness, mechanical elasticity, and non-toxicity [22]. Its complete degradation lasts for approximately two years [23], although it significantly depends on the porosity, molecular weight, and surface area [24]. PCL has been the subject of numerous investigations existing in the literature that are connected to medical applications [25,26,27,28]. It has been a component of composites created for use in several tissue engineering applications [29,30,31,32]. PCL objects have been manufactured by various methods, one of which is additive biomanufacturing [33,34,35]. Additionally, according to the literature, vat photopolymerization (VPP) [36], powder bed fusion [37], and material extrusion (MEX) [38] have also been used for the fabrication of PCL-based parts.

Hydroxyapatite (HAp) is a ceramic [39] material characterized by high bioactivity. It is used for purposes that involve drug delivery [40] and plenty of bone substitutes [41,42,43,44,45,46,47,48,49,50,51]. HAp has been 3D printed for bone tissue [52,53] and scaffolds [54,55]. Synthetic HAp is considered a material for transplantation for tissue replacement [56,57,58,59,60,61]. Some of the existing methods capable of synthesizing HAp include sol-gel [62] reverse microemulsion [63,64], hydrothermal [65], microwave-hydrothermal [66], precipitation [67,68], and solid-state reactions [49].

Tissue engineering scaffolds have been 3D-printed using PCL/HAp composites [69]. Results showed that there was a positive effect on the samples’ tensile and flexural strength ($\sigma_{Β}^{F}$), compared to the neat PCL samples. PCL has also been combined with polylactic acid (PLA) to create composite stents in an effort to create a solution for cardiovascular problems [70]. They have proven to have great potential as biodegradable stents. PCL/HAp composites have been studied in the literature several times in the past in scaffolding for bone tissue engineering [71,72,73,74], bone regeneration [75], biomimetic applications [76], and in vitro and in vivo of bone cells [77]. The behavior has been studied as well [78,79]. Research in PCL/HAp composites in 3D printing is still limited, again focusing on bone tissue engineering applications [80], bio-scaffolds [81], or the use of HAp as a coating in scaffolds made with PCL [82]. Bioplotting the bibliographic research did not reveal any relative research available yet.

In this study, PCL was combined with HAp to create composites of several filler percentages (0.0–8.0% weight concentration, 2.0 step). HAp was chosen as the filler, since it is among the most commonly used bone substitutes, with excellent biocompatibility [83,84]. Additionally, it improves the bond between the material and the living organism, when used in respective applications [85]. Its qualities and characteristics, already described above, constitute HAp a filler with high potential for composites development for use in medical applications. First, the materials were prepared in the appropriate quantities and then fabricated into filaments. This was implemented by an extrusion (thermomechanical) process. The filaments with the composites were later turned into pellets through shredding. These pellets then were the raw material for the 3D fabrication of the specimens through bioplotting. The PCL/HAp samples were tested for mechanical behavior, thermal examination, and rheological analysis, and their structures and morphologies were investigated. In particular, the tests and analyses were related to tensile and flexural response, energy dispersive spectroscopy (EDS), scanning electron microscopy (SEM), Raman spectroscopy, thermogravimetric analysis (TGA), and differential scanning calorimetry (DSC). Moreover, micro-computed tomography (μ-CT) was utilized to determine the dimensional accuracy of the samples, i.e., the deviation between the designed models and the 3D fabricated specimens, as well as to examine their voids and porosity. The bibliographic research did not reveal any similar research on PCL/Hap composites in bioplotting. The characterization of the composites outlined no significant effects on the thermal and other properties of the PCL thermoplastic, while the mechanical performance of the composites was improved. These findings can constitute HAp a valuable filler in biocompatible composites for bioplotting, considering its qualities for medical applications.

## 2. Materials and Methods

**Figure 1:**
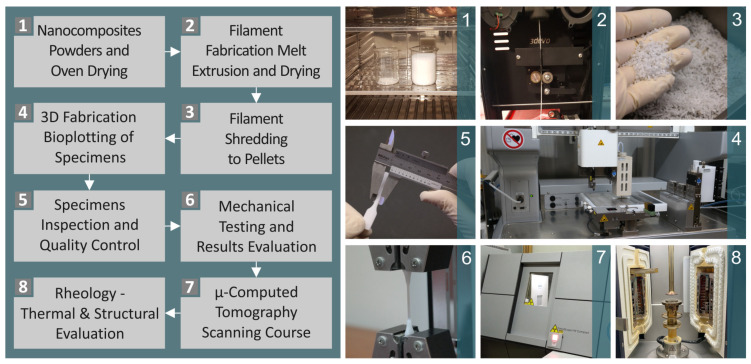
The conducted procedures of the present work namely the (**1**) preparation and drying process of the PCL and HAp raw materials, (**2**) filament extrusion and drying process, (**3**) shredding process of the produced filaments, (**4**) bioplotting for the 3D specimens’ manufacturing, (**5**) quality inspection of the specimens, (**6**) mechanical test of the samples and outcome evaluation, (**7**) μ-CT, and (**8**) rheology, thermal, and structure investigation.

In Figure 1 the guidelines for this work are depicted, from the preparation procedures to the testing and appraisal of the results. The materials in their initial raw form were first placed in an oven for drying (Figure 1(1)) before being supplied to an extruder for the production of the filaments by the melt extrusion method. The produced filaments were then placed to dry in an oven (Figure 1(2)). The filaments were then shredded into pellets (Figure 1(3)), which were then utilized for the 3D fabrication of specimens through bioplotting (Figure 1(4)). The manufactured specimens were inspected and quality controlled (Figure 1(5)) before mechanical testing and evaluation of the results (Figure 1(6)). Moreover, a μ-computed tomography scanning course was conducted (Figure 1(7)), along with rheology, thermal, and structural evaluations (Figure 1(8)).

### 2.1. Material Information

The material polycaprolactone (PCL) employed for the conduction of the present work was sourced from iTVP Denkendorf (Denkendorf, Germany). The material was in medical grade, with an inherent viscosity of about 1.9 dL/g ± 0.3 dL/g and a melting point of around 57.5 °C ± 2.5 °C. The molecular weight of PCL is not provided by the manufacturer in the datasheet. The molecular weight was calculated to be 114.14 g/mol from the PCL formula of this specific grade, which is provided in its datasheet. The material of hydroxyapatite (HAp) (Ca_5_(OH)(PO_4_)_3_) was supplied in white color and powder form (particles in micro-scale), from Sigma-Aldrich Chemie GmbH (Taufkirchen, Germany). It has a molecular weight of 1004.6 g/mol, according to the supplier. The particle size of HAp is 2.5 μm. This was verified in the SEM images of the HAp powder shown below.

### 2.2. Composite Preparation, Mixtures, Filaments and Pellets, and Testing of Filaments

There were five mixtures composed of PCL and HAp, namely 0.0–8.0 wt. % with a 2.0 step. The authors selected this small content increment for the filler to have a more detailed overview of the effect of the filler. At the same time, they wanted to locate the percentage in which the mechanical properties started to decline, indicating the beginning of saturation of the filler in the matrix. First, the PCL matrix material and the HAp filler, with no other additives, were thoroughly blended, utilizing a mixer of high wattage for approximately 20 min at 4000 rpm. The composites were then dried before extrusion into the filaments. A Noztek (by the Noztek company, located in Shoreham-by-Sea, United Kingdom) was utilized for the extrusion procedure. Then, a 3devo shredder (by the 3devo B. V. company, located in Utrecht, The Netherlands) shredded the filaments to produce pellets.

The filler percentage amplitude was determined based on mechanical tests conducted on samples in which the filler percentage was gradually increased. This increase was terminated when the mechanical behavior was no longer positively affected by the addition of the filler, as this decrease can be attributed to the saturation phenomena of the HAp filler in the composites. Such phenomena lead to a decreased mechanical response [86,87].

It should be mentioned that the filaments were monitored in terms of their diameter during production, while they were also mechanically tested in terms of their tensile strength ($\sigma_{Β}^{Τ}$) and tensile modulus of elasticity ($E^{T}$) before being cut by shredding. The diameter was inspected using a digital caliper and random spots were measured during extrusion. Mechanical property information on the filament was collected using an Imada MX2 device (by the company Imada Inc., located in Northbrook, Illinois, United States). The results are presented in the Supplementary Information of this study (Figure S2). The tensile experiments on the filaments to assess their mechanical performance were carried out for completeness and to qualitatively correlate the findings with the samples produced with bioplotting.

### 2.3. Manufacturing of Three-Dimensional Printing Samples

The formation of pellets was followed by the production of the desired 3D-P specimens by bioplotting using an EnvisionTEC developer bio-plotter (Envisiontec GmbH, Gladbeck, Germany). The software that accompanied the procedure was Perfactory RP software (v. 1.30, Envisiontec GmbH, Gladbeck, Germany). The specimens in their digital form were turned into slices of the desired thickness and later, the Perfactory RP v. 3 (Envisiontec GmbH, Gladbeck, Germany) produced data were read by the Visual machine software, with the aim of determining the most suitable printing parameters for the extruder. These parameters are available in the Supplementary Information for this study (Figure S1).

### 2.4. Morphological and Elemental Examination of the Parts

**Figure 2:**
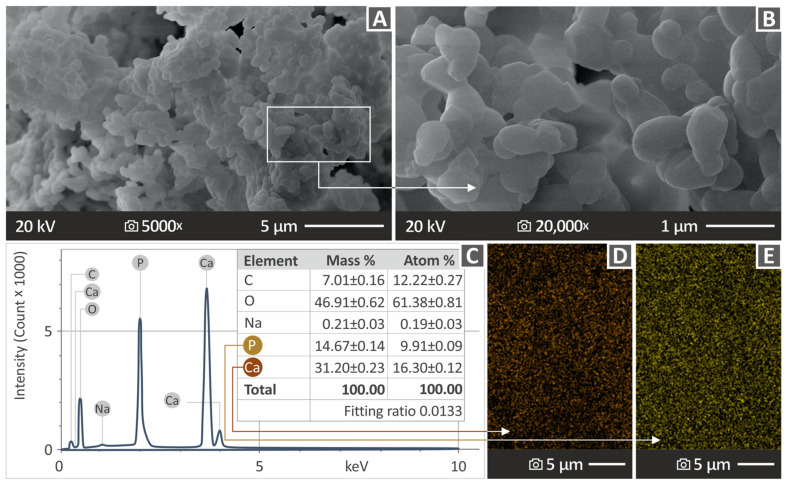
Outcome derived from the SEM and EDS examination of HAp material. (**A**,**B**) SEM pictures at 5000× and 20,000×, respectively, (**C**) EDS analysis indicating the elements found in the chemical composition of HAp, and (**D**,**E**) EDS mapping images showing the dispersion of Ca and P elements, respectively.

SEM and EDS were conducted employing an SEM JSM-IT700HR field emission device (by the Jeol Ltd. Company, located in Tokyo, Japan) for both the HAp raw material and the fabricated specimens. Figure 2A and Figure 2B contain the SEM images of the HAp material at two different magnifications, 5000× and 20,000×, respectively. Figure 2C shows an analysis of the chemical composition of the materials using EDS, along with a board indicating the levels that were measured for each chemical element. Figure 2D and Figure 2E show the EDS mapping images, which indicate the dispersion of Ca and P, respectively. Notably, the concentrations of O, Ca, and P were found to be high, which could be expected because of the chemical formula constituting the HAp material.

### 2.5. Raman Spectral Evaluation

A Raman Spectrometer model named LabRAM HR (by the company HORIBA Scientific, located in Kyoto, Japan) was employed to acquire the Raman spectra. The methodology followed and the parameters used were based on the literature and they presented in the Supplementary Materials of the research [88].

### 2.6. Conduction of Thermal Analysis

Thermogravimetric analysis was conducted on the PCL/HAp composite samples using a Perking Elmer Diamond device (Perking Elmer Diamond, Waltham, MA, USA). The structural integrity was revealed at temperatures between 30 °C and 550 °C. The heat flow in relation to the temperature was also presented in a graph as a part of the differential scanning calorimetry, between the temperature range of −70 °C and 110 °C. DSC investigations were taken on a Discovery Series DSC-25 apparatus (by the TA Instruments company, located in New Castle, DE, USA) equipped with a Refrigerated Cooling System model RSC-90. Both DSC and TGA measurements were taken on an N_2_ gas (inert) atmosphere.

### 2.7. μ-CT Analysis

For microcomputed tomography, the Micro Focus 225 kV CT-scanner model named Tomoscope HV Compact by the company Werth Messtechnik GmbH, located in Gießen, Germany) was employed. The data were analyzed utilizing the software platform VG Studio MAX 2.2 by the Volume Graphics GmbH company, located in Heidelberg, Germany. A micro-focal X-ray tube of 225 kV and a 1024 × 1024 pixel detector was part of the procedure.

### 2.8. Mechanical Equipment and Settings

Tensile experiments were performed using an Imada MX2 machine by the company Imada Inc., located in Northbrook, IL, USA), following the ASTM D638-02a standard [89] (type V specimens, with 3.2 mm height). The flexural tests were carried out in the same machine as the tensile tests, set for three-point bending experiments and at an elongation speed of 10 mm/min and a 52 mm support clearance. The tests were carried out in accordance with the ASTM D790 standard [90]. Please see the Supplementary Materials of the study for further information.

## 3. Results

### 3.1. Raman Spectroscopy and Spectral Differences

**Figure 3:**
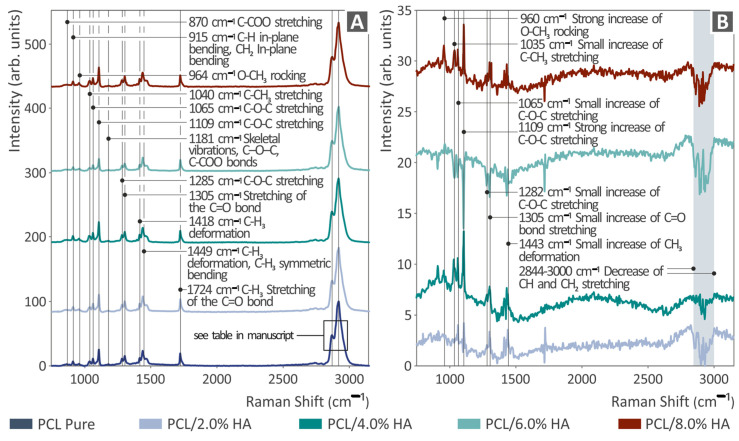
Raman analysis outcome of PCL/HAp (0.0–8.0 wt. %). (**A**) Raman spectra and (**B**) Raman spectral differences.

Figure 3A illustrates the Raman spectral profiles of unfilled PCL and PCL/HAp compounds at all weight percentages (0.0–8.0%). Figure 3B shows the Raman spectral differences of the unfilled PCL and PCL/HAp compositions for all weight percentages (0/0%, 2.0%, 4.0%, 6.0%, and 8.0%) after subtracting the neat PCL. As shown in Figure 3B, the addition of HAp to PCL increased the intensity of the 960, 1035, 1065, 1109, 1282, 1305, and 1443 cm^−1^ Raman bands in all of the PCL/HAp samples. Additionally, there was a decrease in the intensity of the spectral band between 2844 and 3000 cm^−1^. This information is presented in Table 1. The peaks presented in Figure 3 are also presented in table form in the Supplementary Data of the research and are documented in the literature [91,92,93,94,95].

### 3.2. Thermal Characterization

**Figure 4:**
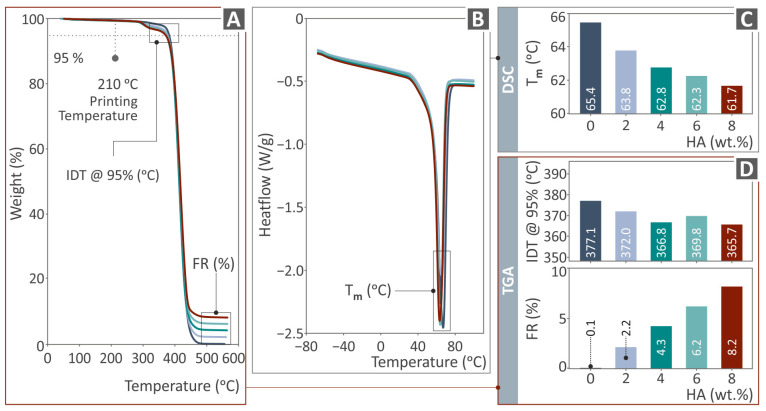
Thermal performance outcome of PCL/HAp (0.0–8.0 wt. %) samples, namely the (**A**) weight as to temperature graphs (TGA), (**B**) heat flow as to temperature graphs (DSC), (**C**) Tm temperature bars versus HAp quantity regarding DSC, and (**D**) FR and IDT values in bars versus HAp quantity, regarding TGA. Each color in the graphs refers to a different composite in terms of HAp content (as shown in **C**,**D**).

Figure 4 presents the results derived after TAG and DSC were performed for all the PCL/HAp composite samples and unfilled PCL. These are the weight vs. the temperature graphs (Figure 4A) and the final residue (FR) and initial decomposition temperature (IDT) levels in the bars (Figure 4D), with regard to TGA, as well as the heat flow as to temperature curves (Figure 4B) and the Tm levels (Figure 4C). It is noticeable from the TGA graphs that as the HAp additive quantity rises, the weight loss declines at temperatures above 450 °C. The introduction of the HAp negligibly alters the response of the PCL thermoplastic to temperature, while the FR agrees with its content in the composites. As can be seen, the temperature amplitude during the DSC examination ranged between approximately −70 °C and 90 °C. Tm levels decreased as the filler percentage decreased, showing an effect on this typical temperature property of the PCL thermoplastic due to the HAp additive. Moreover, the local minimum heat flow value did not appear until the temperature reached approximately 65 °C.

### 3.3. Viscosity and MFR (Rheological Properties)

**Figure 5:**
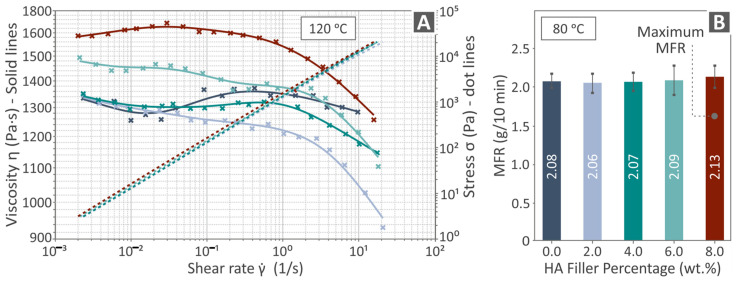
Rheological results of PCL/HAp (0.0–8.0 wt. %) composite samples, namely the (**A**) viscosity vs. shear rate graphs in solid lines and stress vs. shear rate graphs in dot lines as well as the (**B**) MFR vs. HAp quantity bars. Each color in the graphs refers to a different composite in terms of HAp content (as shown in **B**).

In Figure 5, the rheology analysis outcome is presented by possessing viscosity and stress as a shear rate graph (Figure 5A), as well as bars of the MFR (g/ 10 min) levels (Figure 5B) of all PCL/HAp (0.0–80. wt. %) composites. Viscosity measurements were taken at 120 °C and MFR at 80 °C. As the stress increased, the viscosity gradually decreased. Neat PCL shows a similar viscosity graph with two of the composites having median HAp content. The composite with the lower HAp content showed slightly lower viscosity with the increase in the shear rate compared to the unfilled PCL. The higher-loaded composite showed higher viscosity among all the materials tested. Among the composites, the increase in the HAp increased the viscosity. Additionally, it should be mentioned that the MFR levels did not seem to be affected by the increase in filler percentage. Five measurements were taken in each case and the average value and deviation are presented in Figure 5B. MFR measurements were taken in accordance with the ASTM D1238-13 standard [96].

### 3.4. Mechanical Response of 3D-Printed Examples

**Figure 6:**
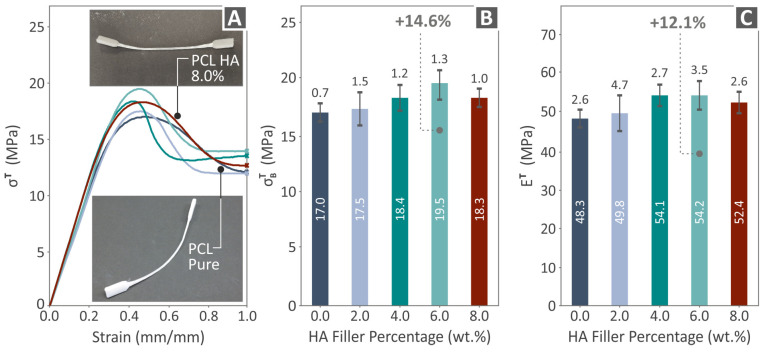
Tensile experiment results on the PCL/HAp (0.0–8.0 wt. %) examples: (**A**) tensile stress to strain graphs and two pictures of PCL/HAp 8.0 wt. % and PCL pure specimens’ condition after the testing, (**B**) $\sigma_{Β}^{Τ}$ vs. HAp quantity, and (**C**) $E^{T}$ vs. HAp filler percentage. Each color in the graphs refers to a different composite in terms of HAp content (as shown in **B**,**C**).

**Figure 7:**
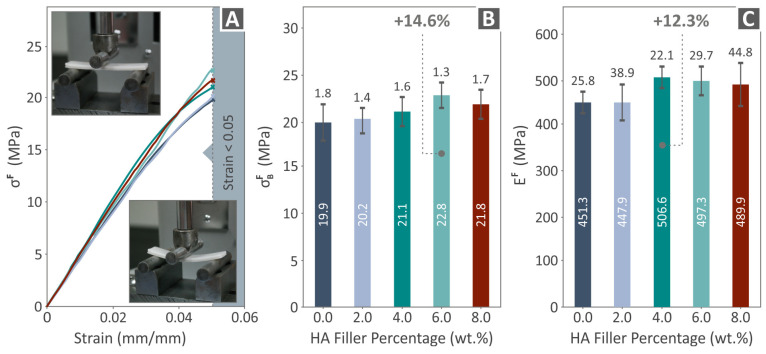
Flexural outcome resulting from the tests conducted on the PCL/HAp (0.0–8.0 wt. %) specimens: (**A**) flexural stress to strain graphs and two pictures of a random specimen’s condition before and after the testing, (**B**) $\sigma_{Β}^{F}$ vs. HAp quantity, and (**C**) $E^{F}$ vs. HAp filler percentage. Each color in the graphs refers to a different composite in terms of HAp content (as shown in **B**,**C**).

**Figure 8:**
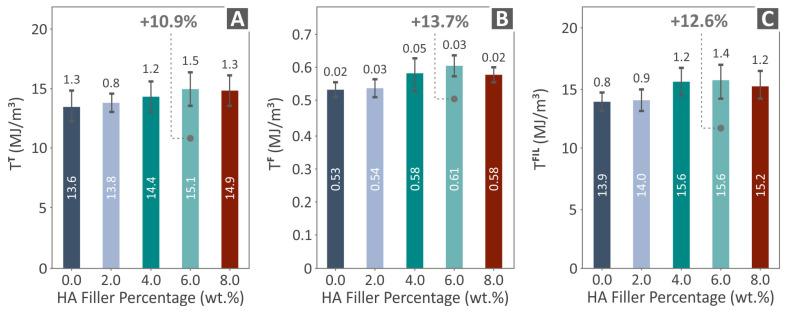
Results from the tests conducted on PCL/HAp (0.0–8.0 wt. %) samples regarding (**A**) tensile toughness, (**B**) flexural toughness, and (**C**) $T^{FIL}$ vs. HAp filler percentage.

The outcome acquired from the mechanical testing of PCL/HAp (0.0–8.0 wt. %) examples can be seen in Figure 6, Figure 7 and Figure 8. Figure 6 presents the outcome after tensile testing, which includes tensile stress-strain graphs (Figure 6A), $\sigma_{Β}^{Τ}$ levels (Figure 6B), and $E^{T}$ levels (Figure 6C). In Figure 6A, the two images show the condition of a random PCL//HAp 8.0 wt. % and a PCL pure tensile specimen, after being tested. Both the $\sigma_{Β}^{Τ}$ and the $E^{T}$ maximum values were detected in PCL/HAp 6.0 wt. % (14.6% and 12.1% improved compared to neat PCL, respectively). As shown, the specimens did not fail in the tensile test after 100% strain and up to the maximum displacement the machine can reach. Therefore, showing the graph beyond 100% strain would not provide any valuable information (the curve was almost parallel to the X-axis line up to the termination of the experiment).

Figure 7 shows the flexural results, including flexural stress to strain graphs (Figure 7A), $\sigma_{Β}^{F}$ levels (Figure 7B), and flexural modulus of elasticity ($E^{F}$) levels (Figure 7C). Figure 7A shows the images acquired during the flexural experiments of a randomly chosen example. The $\sigma_{Β}^{F}$ of PCL/HAp 6.0 wt. % was found to have the highest value over pure PCL, by 14.6%. The most improved levels of $E^{F}$ were detected at PCL/HAp 4.0 wt. %, by being 12.3% over unfilled PCL. Again, the experiment was terminated at 5% without the specimens having failed, in accordance with the specifications of the ASTM D790 [90] international standard.

Figure 8 presents the results related to the tensile (Figure 8A) and flexural toughness (Figure 8B) of the examples as well as the filament toughness (Figure 8C). The composite that presented the highest values in all three cases was PCL/HAp 6.0 wt. %, by being 10.9%, 13.7%, and 12.6% above unfilled PCL, respectively.

### 3.5. Tomography Results of 3D-Bioplotted Specimens

**Figure 9:**
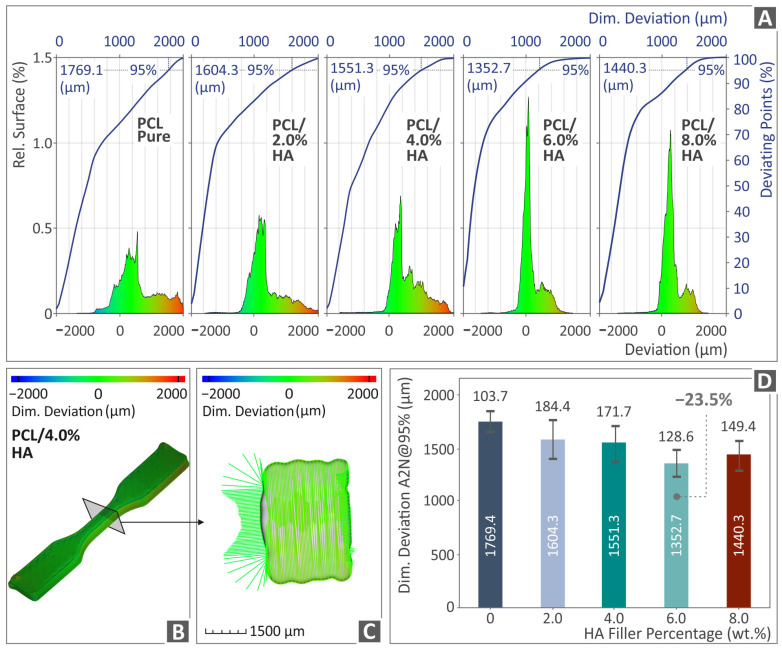
μ-CT outcome of the dimensional deviation, namely the (**A**) graphs of reluctant surface and deviating point vs. deviation of PCL/HAp (0.0–8.0 wt. %) samples, (**B**,**C**) dimensional deviation outcome after the conduction of color-coding mapping on a PCL/HAp 4.0 wt. % tensile specimen, and (**D**) A2N dimensional deviation vs. HAp filler percentage bars of the PCL/HAp (0.0–8.0 wt. %) samples.

**Figure 10:**
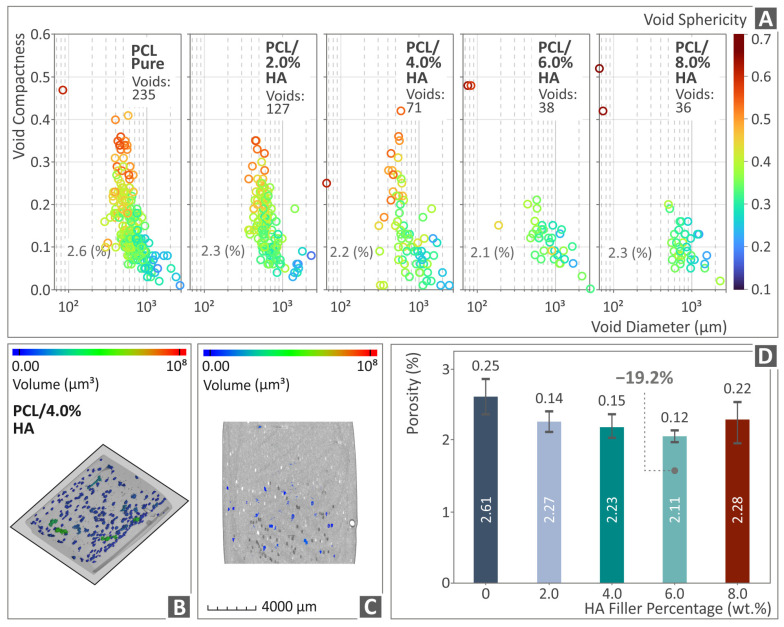
Micro-CT results on the porosity, namely the (**A**) graphs of void compactness and void sphericity vs. void diameter of PCL/HAp (0.0–8.0 wt. %) samples, (**B**,**C**) porosity of a PCL/HAp 4.0 wt. % sample through color-coding mapping, and (**D**) porosity vs. HAp filler percentage bars of the PCL/HAp (0.0–8.0 wt. %) samples.

Figure 9 and Figure 10 depict the outcome of the μ-CT scanning of the PCL/HAp composite samples with regard to the dimensional deviation and porosity, respectively. Figure 9A shows the relative surface detected between the produced and the originally designed PCL/HAp (0.0–8.0 wt. %) specimens in graphs. Figure 9B, C presents a color-coded created image of a random PCL/HAp 4.0 wt. % tensile specimen’s dimensional deviation, while Figure 9D is a presentation of all the PCL/HAp (0.0–8.0 wt. %) composites’ dimensional deviation levels. The composite that was highlighted as the one with the highest levels in relation to unfilled PCL was the PCL/HAp 6.0 wt. %, by being measured 23.5% lower than PCL pure (in dimensional deviation, thus better geometrical accuracy).

Figure 10A presents the compactness and void sphericity versus the diameter of PCL/HAp (0.0–8.0 wt. %) composite samples. Figure 10B, C is a presentation of a random PCL/HAp 4.0 wt. % sample’s porosity through a color-coded mapping. Figure 10D shows the porosity levels of all PCL/HAp (0.0–8.0 wt. %) compounds, with the PCL/HAp 6.0 wt. % being the composite presenting the greatest porosity behavior, by being 19.2% lower than unfilled PCL (less porosity percentage).

### 3.6. Three-Dimensional-Printed Samples’ SEM Morphological Analysis

**Figure 11:**
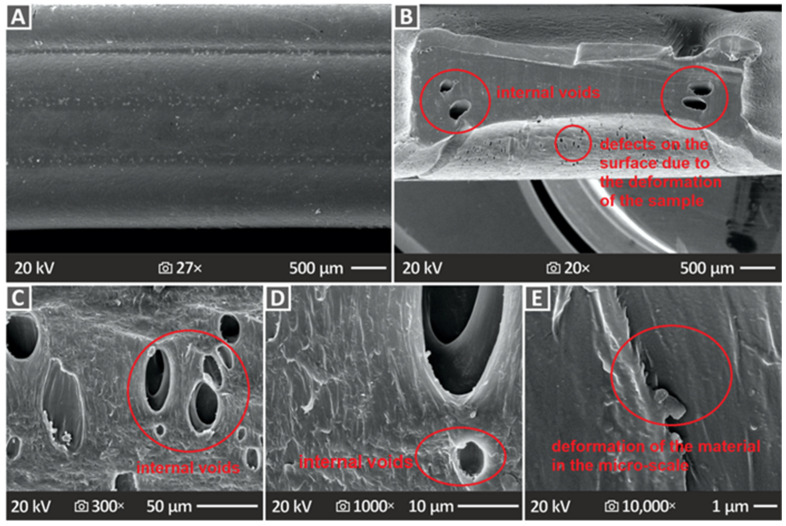
SEM depictions of PCL pure specimens, namely the (**A**) side surface in 27× magnification and (**B**–**E**) cross-section surface in 20×, 300×, 1000×, and 10,000× magnifications, respectively.

**Figure 12:**
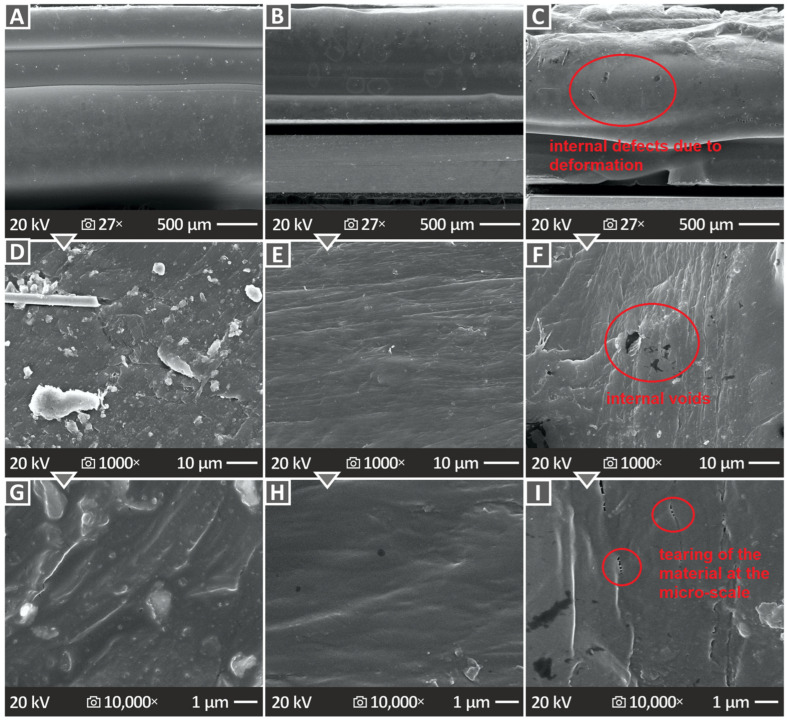
SEM pictures of PCL/HAp 2.0, 4.0, and 8.0 wt. % specimens’ (**A**–**C**) side surfaces in 27× respectively, (**D–F**) cross-section surfaces in 1000×, and (**G**–**I**) 10,000× correspondingly.

Figure 11 and Figure 12 show the SEM images of not only the lateral but also the cross-section surfaces at the neck formed during the tensile experiment. Figure 11 shows pure PCL images, namely, the side surface at 27× magnification (Figure 11A), as well as the cross-section surface at 20×, 300×, 1000×, and 10,000× (Figure 11B–E, respectively). The side surface indicates a large surface without defects; however, the cross-section surfaces reveal many pores and voids of large size. To derive the cross-section images, the samples were cut at the neck area. An intense neck was formed on the samples during the tensile experiment, with a large decrease in the cross-section of the samples, as they were constantly deformed up to the termination of the test, without failure, as mentioned above. This cross-section decrease is easily visible in the SEM images. The internal structure of the samples seems to be solid; the 3D printing structure is not easily distinguishable, yet they are in specific areas on the sides of the cross-section large voids.

On the other hand, Figure 12 shows SEM images of the PCL/HAp 2.0, 4.0, and 8.0 wt. % composite samples’ side surface in 27× magnification (Figure 12A–C), as well as cross-section surfaces (images taken as explained above) magnified in 1000× (Figure 12D–F) and 10,000× (Figure 12G–I), correspondingly. In contrast to the unfilled PCL results, PCL/HAp 2.0, 4.0, and 8.0 wt. % composite samples’ SEM images did not present as many pores and voids, which comes into agreement with the porosity results derived from the μ-CT scanning.

## 4. Discussion

**Figure 13:**
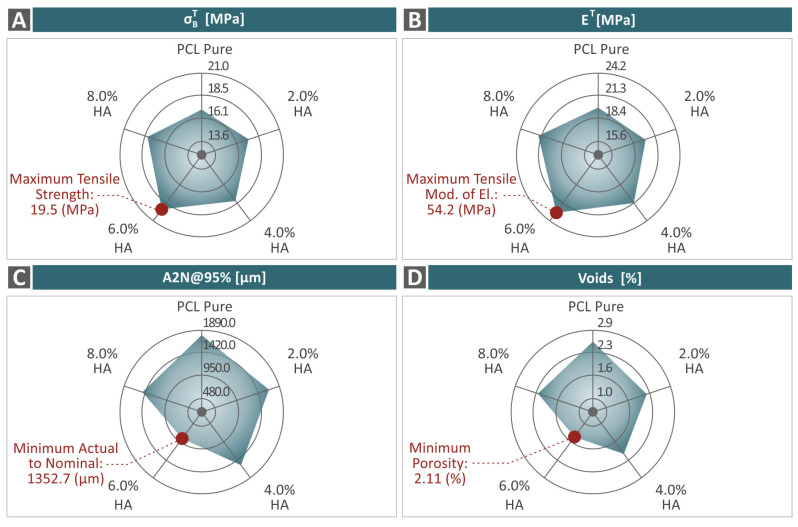
Spider graphs presenting a summarization of the outcome derived from the tests on the PCL/HAp (0.0–8.0 wt. %) samples, with regard to (**A**) $\sigma_{Β}^{Τ}$, (**B**) $E^{T}$, (**C**) A2N95%, and (**D**) voids.

PCL was combined with various concentrations of HAp to produce composites in filaments, pellets, and then specimen forms suitable for investigation in bioplotting 3D printing. Thermal, rheological, mechanical, morphological, and structural investigations possessed results that revealed the reinforcing effect of HAp on the performance of PCL. All the composite samples exhibited enhanced behavior in relation to the unfilled PC. Figure 13 is a summarization of $\sigma_{Β}^{Τ}$, $E^{T}$, A2N, and the voids regarding the PCL/HAp 0.0–8.0 wt. % composite samples. The maximum and minimum values, distinguished from the results, are also highlighted regarding the $\sigma_{Β}^{Τ}$ and $E^{T}$, as well as the A2N and voids, respectively.

The composite with the most positively affected behavior was PCL/HAp 6.0 wt. % sample, which was improved in both the flexural and the tensile properties of the specimens. Moreover, the $E^{F}$ of the PCL/HAp 4.0 wt. % was the most improved in comparison to the PCL pure. The $\sigma_{Β}^{Τ}$, $E^{T}$, and $\sigma_{Β}^{F}$ of PCL/HAp 6.0 wt. % increased by 14.6%, 12.1%, and 14.6%, respectively, over pure PCL. The $E^{F}$ increased by 12.3% compared with that of the unfilled PCL. The $E^{T}$ of the PCL/HAp 6.0 wt. % composite was very close to the maximum value achieved by the 4.0 wt. % composite; hence, the 6.0 wt. % was considered the best concentration for HAp in the PCL matrix, for composites prepared with bioplotting.

The reinforcement mechanism by the addition of the HAp particles in the PCL matrix is owed to the interfacial bonding mechanisms between the polymer and the filler. There are different types of interfacial reinforcement methods and mechanisms [97]. The molecular arrangement of the matrix affects its mechanical properties, while the molecular weight affects the flow properties of the polymer [85,98] and the mechanical properties as well [99]. HAp, being a ceramic material with high stiffness and hardness, can improve the mechanical properties of PCL, which is a relatively soft and flexible polymer. When HAp particles are dispersed within the PCL matrix, they act as stress concentrators and facilitate the transfer of applied loads from the softer PCL to the harder HAp particles. This enhancement is due to the rigid nature of HAp particles, which restrict the mobility of PCL chains, leading to an increase in the composite’s modulus. The interaction at the molecular level between PCL and HAp is crucial for the effective reinforcement of the composite. HAp has a surface rich in hydroxyl groups. These hydroxyl groups can interact with the ester groups of PCL through hydrogen bonding. This hydrogen bonding at the interface leads to better adhesion between the two phases, which is critical for efficient load transfer and the overall mechanical performance of the composite [100,101,102,103].

The tensile and flexural toughness of PCL/HAp 6.0 wt. % were increased by 10.9% and 13.7% over pure PCL, while the filament toughness was 12.6% higher than pure PCL in the case of the same composite. The minimum dimensional deviation was again detected at PCL/HAp 6.0 wt. %, being 23.5% lower than unfilled PCL. The same occurred in the case of porosity, with the lowest value (in relation to unfilled PCL) found in the case of PCL/HAp 6.0 wt. %, by 19.2%. These findings in conjunction with the mechanical experiments results can safely lead to a conclusion that the quality properties are affecting and correlated with the mechanical properties of the composites in this case. Overall, the porosity was decreased by the addition of the HAp particles compared to the unfilled PCL matrix. These changes in the porosity of the 3D printing structure with the addition of the HAp particles can be attributed to changes in the rheological behavior of the composites compared to the neat PCL matrix. The addition of the HAp particles in the matrix did not have a significant effect on the rheology, especially in the MFR, still, the viscosity was slightly altered, leading to differences in the 3D printing structure. It should be clarified that herein, composites with improved mechanical performance were developed. Such composites can be used and are compatible with scaffolding applications. The porosity measured in the study refers to the 3D printing structure of the bio-plotted parts. It is not the porosity of the scaffolds that might hinder tissue integration. The porosity of the scaffolds is a parameter that can be adjusted properly on the geometry of the scaffold and according to the requirements of each application.

The SEM images revealed interesting results, which agreed with porosity-derived information. In particular, unfilled PCL, PCL/HAp 2.0 wt. %, PCL/HAp 4.0 wt. %, and PCL/HAp 8.0 wt. % porosity results were confirmed by the SEM images. It can be observed that pure PCL has many pores and voids, while the next composite possessing prominent porosity, considering the SEM images, was the one with 8.0 wt. % filler quantity, as also stated in the porosity results.

Overall, PCL benefitted from the addition of HAp, especially in the case of 6.0 wt. % filler quantity. The weight loss exhibited better behavior as the filler percentage was increased, whereas the MFR was not notably altered by the introduction of HAp. The viscosity was slightly increased in the higher loaded composite, but, overall, the rheological response of the PCL thermoplastic is not highly impacted by the introduction of the HAp powder. Such findings suggest that the 3D printing parameters do not require each composite to be altered to achieve optimum flow and therefore a 3D printing structure with minimum defects and voids. The mechanical behavior, dimensional deviation, and porosity were positively influenced by HAp. This significantly facilitates the scalability and reproducibility of the 3D bioplotting process. Further optimization, when scaling up for clinical applications, can include the determination of the optimal concentration, by micro-adjusting the HAp concentration around the percentage that achieved the best results. Also, the fine adjustment of the extrusion and bioplotting settings would further improve the performance of the produced composites.

The clinical translation of these PCL/HAp composites would require different regulations to be confronted. The bioplotter used operates in a clean room, which can ensure the confrontation of this part of the process with the regulations. Still, there are other aspects that need to be considered, such as the handling of the raw materials and the ready bio-plotted parts, their transfer to the surgical room, their sterilization, and several other aspects. Initially, preclinical studies need to be conducted to achieve regulatory approval and PCL/HAp composites must undergo rigorous preclinical testing, including in vitro and in vivo studies. These tests evaluate the material’s cytotoxicity, genotoxicity, and potential for causing immune responses. Animal studies are often required to assess the performance and safety of the composites in relevant physiological conditions. Then, clinical trials should follow. After successful preclinical testing, clinical trials are necessary. These trials are conducted in phases, starting with small groups of patients to assess safety, followed by larger studies to evaluate efficacy and side effects. The results must demonstrate that the PCL/HAp composites are safe and effective for the intended medical applications. These tests should confront regulations from the respective organizations. For example, the Food and Drug Administration (FDA) in the USA or the European Medicines Agency (EMA) in the EU. Other standards should be followed, such as ISO 10993 [104] for biocompatibility testing. The compatibility with existing surgical techniques should be considered as well. PCL/HAp composites must be compatible with existing surgical techniques to facilitate their adoption by clinicians. Surgeons need to be able to handle the material similarly to other commonly used implants, such as those made from metals or ceramics. The material’s properties, such as flexibility, moldability, and the ability to be shaped or cut during surgery, play a crucial role. The composites should be compatible with standard fixation methods, such as screws, plates, or sutures, depending on the application. For instance, in orthopedic surgery, the composite must integrate well with bone and allow for secure fixation using conventional hardware. All of these were not within the scope of the current research to be investigated. They are steps to be implemented in the future.

Apart from the PCL/HAp composites presented herein, other commonly used scaffolding materials in bone tissue engineering are categorized as metals, natural and synthetic polymers, and ceramics [105,106]. Comparing the mechanical properties of the composites PCL/HAp composites presented herein with other commonly used scaffolding materials in bone tissue engineering from the literature, it was found that PLA with HAp in 3D printing has been tested with the addition of HAp reducing the mechanical properties (the strength of the neat PLA is comparable to the current study) [107]. The strength of poly(lactic-*co*-glycolic acid—PLGA) was reported to be higher than the current study and it is the only polymer among the ones reported herein with higher mechanical performance than the composites of the current study [108]. The mechanical performance of polyurethane is less than half of that reported herein [25]. Polyvinyl alcohol (PVA) also has been reported to have lower mechanical performance, the lowest among the polymers reported [109,110]. Regarding the natural polymers, such as collagen and chitosan, their strength is lower than the current study [111,112].

Biocompatible eco-friendly additives, such as cellulose in different forms, have been investigated as additives in the PCL polymer in bioplotting. A comparison of the reinforcement effect is presented in the following Table 2. All three composites compared are biocomposites, suitable for medical applications. Still, the use and the qualities of HAp differ. As shown in the table, the maximum improvement in the mechanical performance was achieved at similar filler percentages. The improvement was also close between the composites. The cellulose nanofibers [113] achieved better results, with the cellulose nanocrystals [88] being close. The HAp investigated herein showed slightly lower reinforcement efficacy than the two cellulose forms; still, the differences are not that high. Overall, it should be noted that the research in bioplotting is still limited. The different operation principle of the method justifies the need for such research efforts.

By comparing the current study materials with the literature, it can be noted that PLA is easily synthesized from circular economy resources and is a versatile biopolymer [114]. PLGA has excellent biocompatibility, especially for bone regeneration promotion, it is FDA-approved for clinical applications, and it has adjustable molecular weight, water solubility, and crystallinity tunable by changing the hydroxylation degree. Polyurethane has remarkable mechanical properties. PVA is suitable for manufacturing implants with various characteristics such as the porosity shape and degradation rate. Collagen is chemically modifiable and non-biotoxic, with excellent biodegradability. It is an important part of natural bone organic materials, with excellent biocompatibility. Chitosan has good biodegradability and superior biocompatibility, and it is chemically modifiable [105,106]. Finally, cellulose fibers are used as reinforcing materials for medical implants. They are biodegradable, low-weight, renewable, and cheaper [115,116].

Regarding the environmental impact of producing and disposing of PCL/HAp composites and how it is compared with that of other materials used in similar medical applications, it should be mentioned that this work used a thermomechanical extrusion process to produce the PCL/HAp composites and then the bioplotting process followed. The processes followed did not include the use of any chemical or any harmful materials and no materials were disposed of. So, the environmental impact is expected to be minimal from the process followed in the study. The preparation of composites for medical applications can often include the use of chemical processes, which are more harmful and dangerous to be implemented. PCL/HAp composites generally have a lower environmental impact compared to metals and ceramics, particularly due to their biodegradability and the lower energy requirements for production and disposal. When compared to other biodegradable polymers such as PLA, PCL/HAp composites are competitive. The environmental advantages of PCL/HAp composites make them an attractive option for sustainable medical applications, particularly in scenarios where biodegradability is a key consideration.

Regarding the limitations of the research, the findings are not generalizable to other polymers or biocompatible fillers due to differences in material properties, biocompatibility, and tissue interactions. The interaction between the filler and the matrix differs, as the literature instructs. Addressing these limitations requires targeted research that focuses on understanding and optimizing the unique characteristics of alternative materials. Also, the composite preparation method can affect the results, so this cannot be generalized as well. Limitations can arise from aspects not considered in the study, such as the degradation of materials through time and the confrontation with the regulations. Such issues cannot be estimated at this point in the research.

## 5. Conclusions

The study conducted herein employed PCL and HAp in their raw form to convert them into filaments, which were later turned into pellets and supplied the 3D bioplotting of the specimens. The filler loadings began at 0.0 wt. % and reached 8.0 wt. %, having a step of 2.0.

A composite of PCL/HAp 6.0 wt. % presented better reinforced mechanical behavior, dimensional deviation, and porosity results. In particular, the $\sigma_{Β}^{Τ}$ and $E^{T}$ were improved by 14.6% and 12.1% in relation to pure PCL. The respective flexural properties were improved by 14. 6% in the case of PCL/HAp 6.0 wt. %, while the $E^{F}$ was mostly improved in the case of PCL/HAp 4.0 wt. % by 12.3%, above the properties of pure PCL.

Moreover, the tensile and flexural toughness of the samples, as well as the filaments’ toughness, also showed their greatest improvement at PCL/HAp 6.0 wt. %, by being 10.9%, 13.7%, and 12.6% higher than unfilled PCL, respectively. Additionally, the porosity and dimensional deviation of the same composite were lower than those of pure PCL by 23.5% and 19.2%, respectively. Future work could include an investigation of different filler percentages and mechanical properties. Additionally, future aspects can include the long-term stability of the scaffolds in a biological environment and the potential implications for the long-term performance of these materials, particularly in terms of degradation rates and mechanical integrity over time. Also, the compatibility of the composites with the respective regulations for clinical use in the future requires additional research and optimization.

The key findings are summarized as follows:

- The introduction of HAp in the PCL matrix did not negatively affect the behavior of the polymer, as the characterization process revealed;
- The 6.0 wt. % composite overall was the optimum loading in the study in terms of mechanical reinforcement;
- There was a connection found between the mechanical performance and both the porosity and dimensional accuracy of the samples;
- The study proposed a biocompatible PCL/HAp composite for bioplotting, with robust mechanical performance, which can be an asset for medical-related applications in tissue engineering and scaffolding.

## Figures and Tables

**Table 1:** Significant Raman peak variations in PCL/ Hap samples from PCL/Pure.

Wavenumber (cm^−1^)	Change	Raman Peak Assignment
960	Gradual increase	Strong increase in O-CH_3_ rocking
1035	Gradual increase	Small increase in C-CH_3_ stretching
1065	Gradual increase	Small increase in C-O-C stretching
1109	Gradual increase	Strong increase in C-O-C stretching
1282	increase	Small increase in C-O-C stretching
1305	increase	Small increase in C=O bond stretching
1443	Increase	Small increase in CH_3_ deformation
2844–3000	Gradual decrease	Decrease in CH and CH_2_ stretching

**Table 2:** Effect of different additives in the PCL polymer in bioplotting.

Increase (%)	Current	Cellulose Nanocrystals [88]	Cellulose Nanofibers [113]
Tensile strength	14.6	19.1	23.8
Flexural strength	14.6	12.6	19.1
Optimum loading (wt. %)	6.0	4.0	6.0

## Data Availability

The original contributions presented in the study are included in the article/Supplementary Material, further inquiries can be directed to the corresponding author.

## Supplementary Materials

The following supporting information can be downloaded at https://www.mdpi.com/article/10.3390/polym16172400/s1: Figure S1: List of the printing parameters set for the manufacturing of the 3D printed specimens, pictures of the tensile, flexural, and impact fabricated samples and their initial design accompanied by the respective dimensions and ASTM standards; Figure S2: (A) Image of the fabricated PCL pure filament and its diameter monitoring results, (B) tensile strength results of all the PCL/HA (0.0–8.0 wt. %) filaments, (C) picture of the fabricated PCL/HA 4.0 wt. % filament and its diameter monitoring results, and (D) tensile modulus of elasticity results of all the PCL/HA (0.0–8.0 wt. %) filaments; and Table S1: Significant Raman peaks and their related assignments from PCL pure. References [117,118,119,120,121,122] are cited in the supplementary materials.
