# Supplementary material for: Biomedical Composites of Polycaprolactone/Hydroxyapatite for Bioplotting: Comprehensive Interpretation of the Reinforcement Course
Source: Polymers (Basel). 2024 Aug 24;16(17):2400. doi: 10.3390/polym16172400 (PMC11396925; doi:10.3390/polym16172400)
Supplement: Supplementary file 1 [file polymers-16-02400-s001.zip › polymers-3167538-supplementary.pdf]

S1. 3D printing settings and manufactured samples

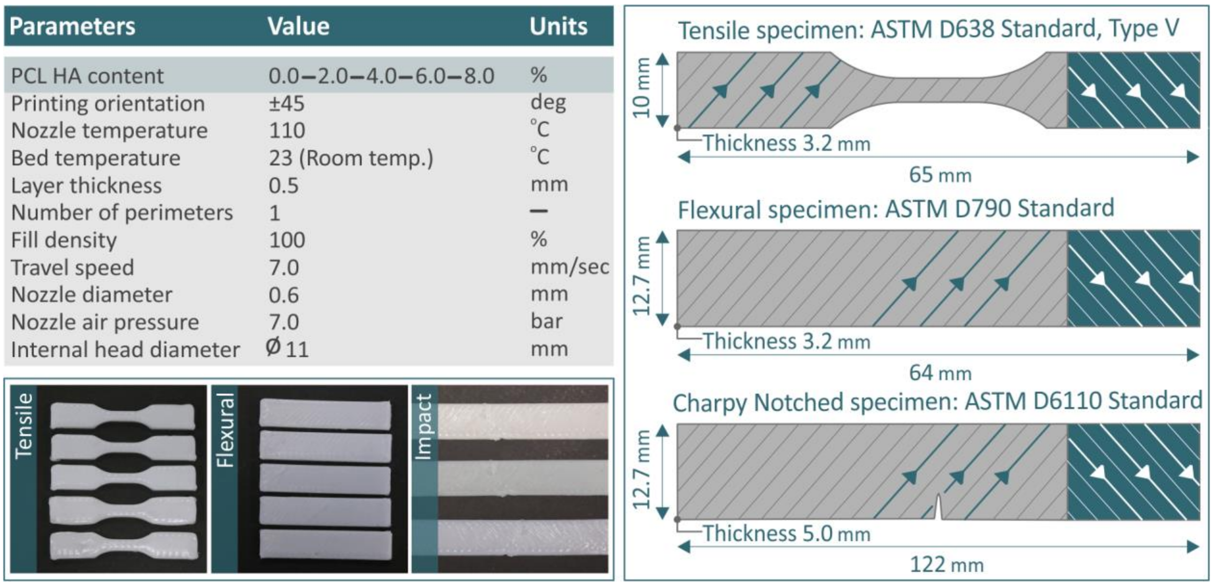

Figure S1. List of the printing parameters set for the manufacturing of the 3D printed specimens, pictures of the tensile, flexural, and impact fabricated samples and their initial design accompanied by the respective dimensions and ASTM standards.

S2. Filament quality evaluation and mechanical performance

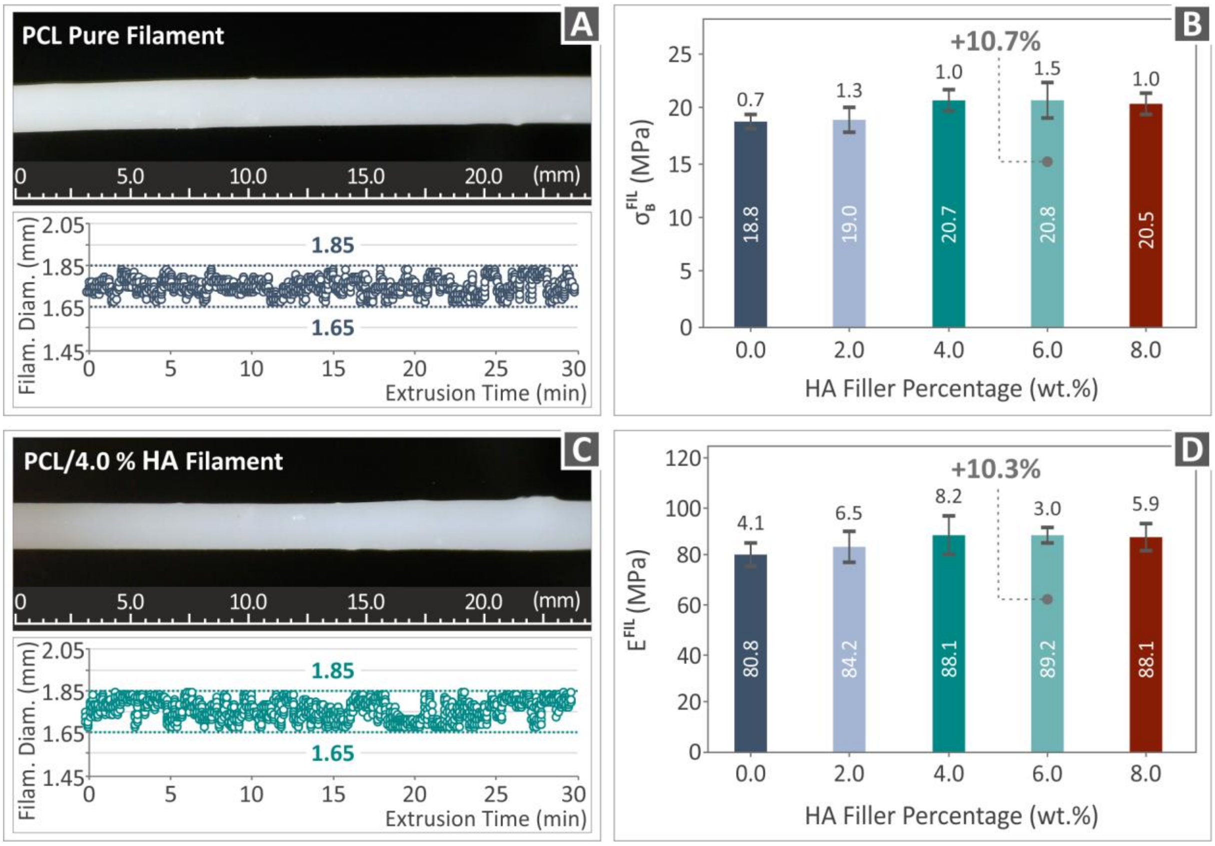

Figure S2. (A) image of the fabricated PCL pure filament and its diameter monitoring results, **(B)** tensile strength results of all the PCL/HA (0.0-8.0 wt. %) filaments, **(C)** picture of the fabricated PCL/HA 4.0 wt. % filament and its diameter monitoring results, **(D)** tensile modulus of elasticity results of all the PCL/HA (0.0-8.0 wt. %) filaments.

S3. Raman Spectral Evaluation

A LabRAM HR Raman Spectrometer (HORIBA Scientific, Kyoto, Japan) was used to acquire the Raman spectra. A 532 nm solid-state laser module was responsible for excitation with a 90 mW maximum output power. There was also an approximately 2 cm^{-1} Raman spectral resolution achieved by a grating with 600 grooves. To deliver a numerical aperture of 0.5, an Olympus objective lens (LMPlanFL N) was employed, and Raman signals were collected. A 50× magnification objective lens operated at a working distance of 10.6 mm. A Neutral Density filter with 5% transmittance was used to limit the laser power, which was measured to be 4.5 mW on the sample. The measurement volume was indicated to be 1.7 μm laterally and 2 μm axially. The collected Raman spectra ranged in amplitude to 40-3900 cm^{-1}, which was accomplished using three optical windows. Each measurement point had a 10 s exposure time and five accumulations. Subsequently, there was a visual inspection of the irradiated areas to be assured of discoloration or degradation (observed as a result of laser irradiation), which was absent.

The Raman raw data were processed using LabSpec software (HORIBA, Kyoto, Japan). The same methodology was employed for every acquired spectrum processing: a) Cosmic rays were removed; b) signal denoise with 5 points kernel; c) background removal using a 7th-grade polynomial; d) spectra were recalibrated by the 2917 cm^{-1} peak and e) spectra were normalized by the maximum peak [1]. In Table S1, the related Raman peaks from the pure PCL sample are extracted from the literature, along with their reference.

Table S1. Significant Raman peaks and their related assignments from PCL pure.

Wavenumber (cm^{-1})	Strength	Raman peak assignment
870	Small	C-COO stretching [2]
915	Medium	C-H in-plane bending [3] ; CH_2 In-plane bending [4]
964	Small	O-CH_3 rocking [5]
1040	Medium	C-CH_3 stretching [2]
1065	Medium	C-O-C stretching [2]
1109	Strong	C-O-C stretching [2]
1181	Small	Skeletal vibrations, C–O– C, C-COO bonds [5–7]
1285	Medium	C-O-C stretching [2]
1305	Medium	Stretching of the C=O bond [8]
1418	Medium	C-H_3 deformation [3]
1449	Strong	C-H_3 deformation [3,5,7]; C-H_3 symmetric bending [2,3,9];
1724	Strong	Stretching of the C=O bond [8,10,11]
2870	Strong	C-H_2 symmetric stretching [6]; C-H symmetric stretching [12]
2919	Strong	CH_2 asymmetric stretching [6]

References

1. Vidakis, N.; Petousis, M.; Michailidis, N.; David, C.; Mountakis, N.; Papadakis, V.; Sfakiotakis, E.; Sagris, D.; Argyros, A. Optimization of Cellulose Nanocrystal (CNC) Concentration in Polycaprolactone Bio-Composites for Bio-Plotting: A Robust Interpretation of the Reinforcement Mechanisms. *Cellulose* **2024**, doi:10.1007/s10570-024-05851-7.
2. Lin, Z.; Guo, X.; He, Z.; Liang, X.; Wang, M.; Jin, G. Thermal Degradation Kinetics Study of Molten Polylactide Based on Raman Spectroscopy. *Polym Eng Sci* **2021**, *61*, 201–210, doi:10.1002/pen.25568.
3. Stuart, B.H. Temperature Studies of Polycarbonate Using Fourier Transform Raman Spectroscopy. *Polymer Bulletin* **1996**, *36*, 341–346, doi:10.1007/BF00319235.
4. Camerlingo, C.; Zenone, F.; Delfino, I.; Diano, N.; Mita, D.G.; Lepore, M. Investigation on Clarified Fruit Juice Composition by Using Visible Light Micro-Raman Spectroscopy. *Sensors* **2007**, *7*, 2049–2061, doi:10.3390/s7102049.
5. Veluthandath, A. V.; Bisht, P.B. Identification of Whispering Gallery Mode (WGM) Coupled Photoluminescence and Raman Modes in Complex Spectra of MoS 2 in Polymethyl Methacrylate (PMMA) Microspheres. *J Lumin* **2017**, *187*, 255–259, doi:10.1016/j.jlumin.2017.03.031.
6. Makarem, M.; Lee, C.M.; Kafle, K.; Huang, S.; Chae, I.; Yang, H.; Kubicki, J.D.; Kim, S.H. Probing Cellulose Structures with Vibrational Spectroscopy. *Cellulose* **2019**, *26*, 35–79, doi:10.1007/s10570-018-2199-z.
7. Zimmerer, C.; Matulaitiene, I.; Niaura, G.; Reuter, U.; Janke, A.; Boldt, R.; Sablinskas, V.; Steiner, G. Nondestructive Characterization of the Polycarbonate - Octadecylamine Interface by Surface Enhanced Raman Spectroscopy. *Polym Test* **2019**, *73*, 152–158, doi:10.1016/j.polymertesting.2018.11.023.

8. Gatin, E.; Iordache, S.-M.; Matei, E.; Luculescu, C.-R.; Iordache, A.-M.; Grigorescu, C.; Ilici, R. Raman Spectroscopy as Spectral Tool for Assessing the Degree of Conversion after Curing of Two Resin-Based Materials Used in Restorative Dentistry. *Diagnostics* **2022**, *12*, 1993, doi:10.3390/diagnostics12081993.
9. Resta, V.; Quarta, G.; Lomascolo, M.; Maruccio, L.; Calcagnile, L. Raman and Photoluminescence Spectroscopy of Polycarbonate Matrices Irradiated with Different Energy 28Si+ Ions. *Vacuum* **2015**, *116*, 82–89, doi:10.1016/j.vacuum.2015.03.005.
10. Luiz, B.K.M.; Amboni, R.D.M.C.; Prates, L.H.M.; Roberto Bertolino, J.; Pires, A.T.N. Influence of Drinks on Resin Composite: Evaluation of Degree of Cure and Color Change Parameters. *Polym Test* **2007**, *26*, 438–444, doi:10.1016/j.polymertesting.2006.12.005.
11. Peris-Díaz, M.D.; Łydżba-Kopczyńska, B.; Sentandreu, E. Raman Spectroscopy Coupled to Chemometrics to Discriminate Provenance and Geological Age of Amber. *Journal of Raman Spectroscopy* **2018**, *49*, 842–851, doi:10.1002/jrs.5357.
12. Liu, X.; Zou, Y.; Li, W.; Cao, G.; Chen, W. Kinetics of Thermo-Oxidative and Thermal Degradation of Poly(d,l-Lactide) (PDLLA) at Processing Temperature. *Polym Degrad Stab* **2006**, *91*, 3259–3265, doi:10.1016/j.polymdegradstab.2006.07.004.
